# A third polymorph of *N*,*N*′-bis­(pyridin-2-yl)benzene-1,4-diamine

**DOI:** 10.1107/S1600536811044539

**Published:** 2011-10-29

**Authors:** Barbara Wicher, Maria Gdaniec

**Affiliations:** a Faculty of Chemistry, Adam Mickiewicz University, 60-780 Poznań, Poland

## Abstract

A third polymorph of the title compound, C_16_H_14_N_4_, has been obtained. The mol­ecule adopts a non-planar conformation with an *E* configuration at the two partially double *exo* C N bonds of the 2-pyridyl­amine units. Like in the triclinic form [Bensemann *et al.* (2002 ▶). *New J. Chem.* **26**, 448–456], the recognition process between 2-pyridyl­amine units takes place through formation of a cyclic *R*_2_^2^(8) hydrogen-bond motif, leading to the creation of tapes parallel to [001].

## Related literature

For the structures of the ortho­rhom­bic and triclinic polymorphs of *N*,*N*′-di(pyridin-2-yl)benzene-1,4-diamine, see: Bensemann *et al.* (2002 ▶).
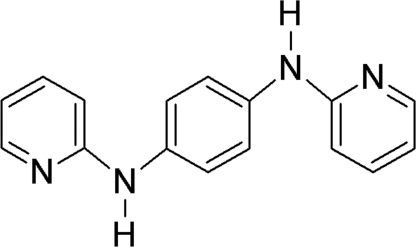


## Experimental

### 

#### Crystal data

C_16_H_14_N_4_
*M* *_r_* = 262.31
Monoclinic, 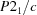

*a* = 7.2534 (2) Å
*b* = 20.8270 (6) Å
*c* = 9.0681 (3) Å
β = 106.746 (4)°
*V* = 1311.79 (7) Å^3^
*Z* = 4
Cu *K*α radiation
μ = 0.65 mm^−1^
*T* = 295 K
0.2 × 0.2 × 0.05 mm

#### Data collection

Oxford Diffraction SuperNova diffractometer
Absorption correction: multi-scan (*CrysAlis PRO*; Agilent, 2010 ▶) *T*_min_ = 0.799, *T*_max_ = 1.000
10413 measured reflections
2398 independent reflections
2221 reflections with *I* > 2/s(*I*)
*R*_int_ = 0.019

#### Refinement

*R*[*F* ^2^ > 2σ(*F* ^2^)] = 0.035
*wR*(*F* ^2^) = 0.094
*S* = 1.07
2398 reflections
181 parameters
H-atom parameters constrained
Δρ_max_ = 0.11 e Å^−3^
Δρ_min_ = −0.19 e Å^−3^

### 

Data collection: *CrysAlis PRO* (Agilent, 2010 ▶); cell refinement: *CrysAlis PRO*; data reduction: *CrysAlis PRO*; program(s) used to solve structure: *SIR2004* (Burla *et al.*, 2005 ▶); program(s) used to refine structure: *SHELXL97* (Sheldrick, 2008 ▶); molecular graphics: *ORTEP-3 for Windows* (Farrugia, 1997 ▶) and *Mercury* (Macrae *et al.*, 2006 ▶); software used to prepare material for publication: *SHELXL97*.

## Supplementary Material

Crystal structure: contains datablock(s) global, I. DOI: 10.1107/S1600536811044539/rz2657sup1.cif

Structure factors: contains datablock(s) I. DOI: 10.1107/S1600536811044539/rz2657Isup2.hkl

Supplementary material file. DOI: 10.1107/S1600536811044539/rz2657Isup3.cml

Additional supplementary materials:  crystallographic information; 3D view; checkCIF report

## Figures and Tables

**Table 1:** Hydrogen-bond geometry (Å, °)

*D*—H⋯*A*	*D*—H	H⋯*A*	*D*⋯*A*	*D*—H⋯*A*
N7—H7*N*⋯N16^i^	0.90	2.25	3.1423 (13)	175
N14—H14*N*⋯N2^ii^	0.90	2.12	3.0141 (14)	175

Symmetry codes: (i) 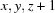; (ii) 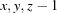.

**Fig. 1.**
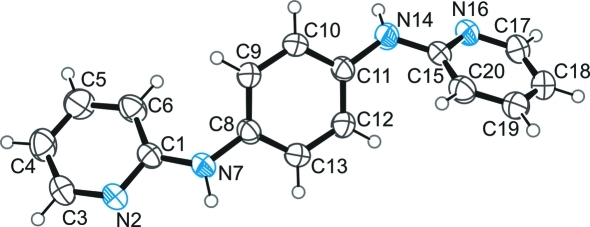
: The asymmetric unit of the title compound with displacement ellipsoids shown at the 50% probability level.

**Fig. 2.**
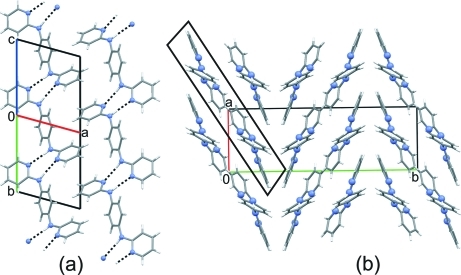
: Crystal packing in the monoclinic polymorph of the title compound: (a) a pair of hydrogen bonded tapes extended along [0 0 1] and (b) herring-bone packing of the pairs of tapes (one pair is show with a black rhomboid). Hydrogen bonds are shown with dashed lines.

## supplementary crystallographic information

### Comment

The compounds bearing two 2-pyridylamine groups separated by linkers can adopt either *E,E*, *Z,Z* or *E,Z* forms depending on the configuration of the partially double *exo* C≐ N bond of the 2-pyridylamine unit. In crystals the molecules in the *E, E* form tend to build one-dimensional networks *via R*^2^_2_(8) synthons generated between self-complementary 2-pyridylamine groups. In turn, the *Z,Z* form generates the C(4) catemer motif that can lead to the formation of one-, two- and three-dimensional frameworks (Bensemann *et al.*, 2002). These compounds are known to exhibit conformational polymorphism and for *N*,*N*'-di(pyridin-2-yl)benzene-1,4-diamine two polymorphic forms were identified. In the orthorhombic form (Pbca, *Z*'=0.5), obtained by crystallization from acetonitrile, the molecules are nonplanar and adopt the *Z,Z* form. Hydrogen bonds between 2-pyridylamine groups generate catemeric motifs that assemble molecules into a two-dimensional framework. In the triclinic *P*1 polymorph, obtained by crystallization from methanol, there are two symmetry independent molecules, each in the *E,E* form and located around inversion center. These molecules form tapes *via* strongly nonplanar *R*^2^_2_(8) motif generated by N—H···N hydrogen bonds (Bensemann *et al.*, 2002).

Recently, during an attempt to cocrystallize *N*,*N*'-di(pyridin-2-yl)benzene-1,4-diamine with pyrazine from 2-butanone, a new monoclinic polymorph of *N*,*N*'-di(pyridin-2-yl)benzene-1,4-diamine was obtained. When crystallization was repeated from 2-butanone without addition of pyrazine the triclinic polymorph was formed.

In the new monoclinic polymorph the molecules adopt the *E,E* form and are assembled into tapes *via* strongly non-planar *R*^2^_2_(8) hydrogen-bond motif. The overall shape of the tapes and their crystal packing are different from the arrangement found in the triclinic polymorph. As shown in Fig. 2a, the hydrogen-bonded tapes extended along [0 0 1] are grouped into pairs, with no specific interactions occurring between the two tapes, and these pairs of tapes are further arranged in a herring-bone manner (Fig. 2b).

The three polymorphs of *N*,*N*'-di(pyridin-2-yl)benzene-1,4-diamine have identical or very similar melting points: 478–479 K for the orthorhombic and triclinic polymorphs and 479 K for the monoclinic form. The calculated crystal densities are also similar: 1.335, 1.314 and 1.328 g cm^-3^ for orthorhombic, triclinic and monoclinic forms, respectively.

### Experimental

*N*,*N*'-Di(pyridin-2-yl)benzene-1,4-diamine was prepared according to the published procedure (Bensemann *et al.*, 2002). *N*,*N*'-Di(pyridin-2-yl)benzene-1,4-diamine (0.03 g, 0.11 mmol) and pyrazine (0.01 g, 0.11 mmol) were dissolved in 5 ml of 2-butanone and placed in a glass vial. After a few days colourless, plate-shaped crystals with a melting point of 479 K were obtained.

### Refinement

H atoms of the N—H groups were located in difference electron-density maps. N—H bond lengths were standardized to 0.90 Å and *U*_iso_(H) values were constrained to 1.2*U*_eq_(N). All other H atoms were initially identified in difference maps but were placed at calculated positions with C—H = 0.93 Å, and were refined as riding on their carrier atoms with *U*_iso_(H) = 1.2*U*_eq_(C).

### Crystal data

**Table:**

C_16_H_14_N_4_	*F*(000) = 552
*M**_r_* = 262.31	*D*_x_ = 1.328 Mg m^−^^3^
Monoclinic, *P*2_1_/*c*	Melting point: 479 K
Hall symbol: -P 2ybc	Cu *K*α radiation, λ = 1.54178 Å
*a* = 7.2534 (2) Å	Cell parameters from 6262 reflections
*b* = 20.8270 (6) Å	θ = 2.1–75.8°
*c* = 9.0681 (3) Å	µ = 0.65 mm^−^^1^
β = 106.746 (4)°	*T* = 295 K
*V* = 1311.79 (7) Å^3^	Plate, colourless
*Z* = 4	0.2 × 0.2 × 0.05 mm

### Data collection

**Table:**

Oxford Diffraction SuperNova diffractometer	2398 independent reflections
Radiation source: Nova Cu X-ray Source	2221 reflections with *I* > 2/s(*I*)
mirror	*R*_int_ = 0.019
ω scans	θ_max_ = 68.2°, θ_min_ = 6.4°
Absorption correction: multi-scan (*CrysAlis PRO*; Agilent, 2010)	*h* = −8→8
*T*_min_ = 0.799, *T*_max_ = 1.000	*k* = −25→25
10413 measured reflections	*l* = −10→10

### Refinement

**Table:**

Refinement on *F*^2^	Primary atom site location: structure-invariant direct methods
Least-squares matrix: full	Secondary atom site location: difference Fourier map
*R*[*F*^2^ > 2σ(*F*^2^)] = 0.035	Hydrogen site location: inferred from neighbouring sites
*wR*(*F*^2^) = 0.094	H-atom parameters constrained
*S* = 1.07	*w* = 1/[σ^2^(*F*_o_^2^) + (0.0478*P*)^2^ + 0.2155*P*] where *P* = (*F*_o_^2^ + 2*F*_c_^2^)/3
2398 reflections	(Δ/σ)_max_ < 0.001
181 parameters	Δρ_max_ = 0.11 e Å^−^^3^
0 restraints	Δρ_min_ = −0.19 e Å^−^^3^

### Special details

**Table:**

Geometry. All e.s.d.'s (except the e.s.d. in the dihedral angle between two l.s. planes) are estimated using the full covariance matrix. The cell e.s.d.'s are taken into account individually in the estimation of e.s.d.'s in distances, angles and torsion angles; correlations between e.s.d.'s in cell parameters are only used when they are defined by crystal symmetry. An approximate (isotropic) treatment of cell e.s.d.'s is used for estimating e.s.d.'s involving l.s. planes.
Refinement. Refinement of *F*^2^ against ALL reflections. The weighted *R*-factor *wR* and goodness of fit *S* are based on *F*^2^, conventional *R*-factors *R* are based on *F*, with *F* set to zero for negative *F*^2^. The threshold expression of *F*^2^ > σ(*F*^2^) is used only for calculating *R*-factors(gt) *etc*. and is not relevant to the choice of reflections for refinement. *R*-factors based on *F*^2^ are statistically about twice as large as those based on *F*, and *R*- factors based on ALL data will be even larger.

### Fractional atomic coordinates and isotropic or equivalent isotropic displacement parameters (Å^2^)

**Table:**

	*x*	*y*	*z*	*U*_iso_*/*U*_eq_	
N2	0.18091 (14)	0.15267 (5)	0.53715 (10)	0.0434 (2)	
N7	0.34929 (14)	0.13813 (5)	0.36249 (10)	0.0453 (2)	
H7N	0.4266	0.1142	0.4380	0.054*	
N14	0.53458 (15)	0.12536 (5)	−0.19925 (11)	0.0521 (3)	
H14N	0.4314	0.1319	−0.2811	0.063*	
N16	0.63876 (14)	0.06275 (5)	−0.36845 (10)	0.0458 (2)	
C1	0.18087 (15)	0.15794 (5)	0.38962 (12)	0.0382 (2)	
C3	0.02222 (18)	0.17053 (6)	0.57328 (14)	0.0500 (3)	
H3	0.0216	0.1663	0.6752	0.060*	
C4	−0.13952 (18)	0.19473 (7)	0.46964 (16)	0.0572 (3)	
H4	−0.2462	0.2071	0.5000	0.069*	
C5	−0.13771 (18)	0.19995 (7)	0.31846 (16)	0.0581 (3)	
H5	−0.2449	0.2161	0.2448	0.070*	
C6	0.02097 (17)	0.18144 (6)	0.27640 (13)	0.0486 (3)	
H6	0.0224	0.1844	0.1744	0.058*	
C8	0.39192 (15)	0.13618 (5)	0.22059 (12)	0.0387 (3)	
C9	0.34861 (17)	0.18587 (5)	0.11355 (13)	0.0435 (3)	
H9	0.2842	0.2220	0.1332	0.052*	
C10	0.40017 (17)	0.18215 (6)	−0.02162 (12)	0.0445 (3)	
H10	0.3685	0.2157	−0.0920	0.053*	
C11	0.49825 (15)	0.12933 (5)	−0.05437 (12)	0.0406 (3)	
C12	0.54745 (16)	0.08080 (5)	0.05517 (13)	0.0425 (3)	
H12	0.6177	0.0457	0.0380	0.051*	
C13	0.49368 (16)	0.08396 (5)	0.18909 (13)	0.0419 (3)	
H13	0.5262	0.0505	0.2597	0.050*	
C15	0.67958 (16)	0.08914 (5)	−0.22815 (12)	0.0418 (3)	
C17	0.7754 (2)	0.02598 (6)	−0.39897 (15)	0.0539 (3)	
H17	0.7477	0.0068	−0.4955	0.065*	
C18	0.9526 (2)	0.01483 (7)	−0.29741 (16)	0.0603 (4)	
H18	1.0408	−0.0122	−0.3228	0.072*	
C19	0.99619 (18)	0.04509 (7)	−0.15578 (15)	0.0574 (3)	
H19	1.1173	0.0402	−0.0855	0.069*	
C20	0.85928 (17)	0.08235 (7)	−0.11991 (14)	0.0506 (3)	
H20	0.8858	0.1028	−0.0249	0.061*	

### Atomic displacement parameters (Å^2^)

**Table:**

	*U*^11^	*U*^22^	*U*^33^	*U*^12^	*U*^13^	*U*^23^
N2	0.0485 (5)	0.0489 (5)	0.0360 (5)	0.0011 (4)	0.0174 (4)	0.0017 (4)
N7	0.0482 (5)	0.0582 (6)	0.0316 (5)	0.0106 (4)	0.0148 (4)	0.0050 (4)
N14	0.0529 (6)	0.0744 (7)	0.0294 (5)	0.0220 (5)	0.0123 (4)	0.0021 (4)
N16	0.0504 (6)	0.0555 (6)	0.0349 (5)	0.0066 (4)	0.0177 (4)	0.0011 (4)
C1	0.0431 (6)	0.0385 (5)	0.0343 (5)	−0.0050 (4)	0.0132 (4)	−0.0027 (4)
C3	0.0539 (7)	0.0573 (7)	0.0456 (6)	−0.0034 (5)	0.0253 (5)	−0.0006 (5)
C4	0.0427 (6)	0.0708 (9)	0.0636 (8)	−0.0016 (6)	0.0242 (6)	−0.0007 (6)
C5	0.0386 (6)	0.0760 (9)	0.0563 (8)	−0.0020 (6)	0.0081 (5)	0.0052 (6)
C6	0.0438 (6)	0.0638 (7)	0.0365 (6)	−0.0053 (5)	0.0090 (5)	−0.0002 (5)
C8	0.0403 (6)	0.0454 (6)	0.0313 (5)	0.0006 (4)	0.0117 (4)	−0.0013 (4)
C9	0.0497 (6)	0.0445 (6)	0.0392 (6)	0.0108 (5)	0.0174 (5)	0.0014 (5)
C10	0.0505 (6)	0.0488 (6)	0.0352 (6)	0.0122 (5)	0.0139 (5)	0.0080 (5)
C11	0.0409 (6)	0.0511 (6)	0.0302 (5)	0.0054 (5)	0.0108 (4)	−0.0014 (4)
C12	0.0465 (6)	0.0415 (6)	0.0411 (6)	0.0078 (5)	0.0150 (5)	−0.0023 (4)
C13	0.0474 (6)	0.0413 (6)	0.0377 (6)	0.0046 (5)	0.0134 (5)	0.0051 (4)
C15	0.0471 (6)	0.0488 (6)	0.0335 (5)	0.0057 (5)	0.0178 (5)	0.0048 (4)
C17	0.0630 (8)	0.0606 (7)	0.0455 (7)	0.0103 (6)	0.0273 (6)	−0.0004 (5)
C18	0.0604 (8)	0.0689 (8)	0.0618 (8)	0.0204 (6)	0.0337 (7)	0.0123 (7)
C19	0.0445 (7)	0.0763 (9)	0.0538 (7)	0.0105 (6)	0.0180 (6)	0.0175 (6)
C20	0.0489 (7)	0.0660 (8)	0.0373 (6)	0.0036 (5)	0.0131 (5)	0.0023 (5)

### Geometric parameters (Å, °)

**Table:**

N2—C3	1.3372 (15)	C8—C13	1.3895 (15)
N2—C1	1.3422 (13)	C8—C9	1.3916 (15)
N7—C1	1.3771 (14)	C9—C10	1.3829 (15)
N7—C8	1.4079 (13)	C9—H9	0.9300
N7—H7N	0.9001	C10—C11	1.3879 (16)
N14—C15	1.3793 (14)	C10—H10	0.9300
N14—C11	1.4148 (14)	C11—C12	1.3898 (16)
N14—H14N	0.9000	C12—C13	1.3799 (15)
N16—C15	1.3385 (14)	C12—H12	0.9300
N16—C17	1.3427 (15)	C13—H13	0.9300
C1—C6	1.3974 (16)	C15—C20	1.3947 (16)
C3—C4	1.3706 (19)	C17—C18	1.3682 (19)
C3—H3	0.9300	C17—H17	0.9300
C4—C5	1.3790 (18)	C18—C19	1.383 (2)
C4—H4	0.9300	C18—H18	0.9300
C5—C6	1.3681 (18)	C19—C20	1.3713 (18)
C5—H5	0.9300	C19—H19	0.9300
C6—H6	0.9300	C20—H20	0.9300
			
C3—N2—C1	117.93 (10)	C8—C9—H9	119.6
C1—N7—C8	127.71 (9)	C9—C10—C11	121.35 (10)
C1—N7—H7N	114.8	C9—C10—H10	119.3
C8—N7—H7N	115.3	C11—C10—H10	119.3
C15—N14—C11	124.37 (9)	C10—C11—C12	117.74 (10)
C15—N14—H14N	115.1	C10—C11—N14	119.28 (10)
C11—N14—H14N	115.1	C12—C11—N14	122.89 (10)
C15—N16—C17	117.13 (10)	C13—C12—C11	120.99 (10)
N2—C1—N7	114.09 (10)	C13—C12—H12	119.5
N2—C1—C6	121.52 (10)	C11—C12—H12	119.5
N7—C1—C6	124.39 (10)	C12—C13—C8	121.32 (10)
N2—C3—C4	124.16 (11)	C12—C13—H13	119.3
N2—C3—H3	117.9	C8—C13—H13	119.3
C4—C3—H3	117.9	N16—C15—N14	115.68 (10)
C3—C4—C5	117.34 (12)	N16—C15—C20	122.22 (10)
C3—C4—H4	121.3	N14—C15—C20	122.07 (10)
C5—C4—H4	121.3	N16—C17—C18	124.33 (12)
C6—C5—C4	120.31 (12)	N16—C17—H17	117.8
C6—C5—H5	119.8	C18—C17—H17	117.8
C4—C5—H5	119.8	C17—C18—C19	117.84 (12)
C5—C6—C1	118.73 (11)	C17—C18—H18	121.1
C5—C6—H6	120.6	C19—C18—H18	121.1
C1—C6—H6	120.6	C20—C19—C18	119.38 (12)
C13—C8—C9	117.73 (10)	C20—C19—H19	120.3
C13—C8—N7	118.69 (10)	C18—C19—H19	120.3
C9—C8—N7	123.44 (10)	C19—C20—C15	118.97 (12)
C10—C9—C8	120.81 (10)	C19—C20—H20	120.5
C10—C9—H9	119.6	C15—C20—H20	120.5
			
C3—N2—C1—N7	−179.60 (10)	C15—N14—C11—C10	157.24 (12)
C3—N2—C1—C6	0.15 (16)	C15—N14—C11—C12	−26.30 (18)
C8—N7—C1—N2	178.25 (10)	C10—C11—C12—C13	2.54 (17)
C8—N7—C1—C6	−1.49 (19)	N14—C11—C12—C13	−173.97 (11)
C1—N2—C3—C4	−0.97 (18)	C11—C12—C13—C8	−1.25 (17)
N2—C3—C4—C5	0.9 (2)	C9—C8—C13—C12	−1.05 (17)
C3—C4—C5—C6	−0.1 (2)	N7—C8—C13—C12	−176.94 (10)
C4—C5—C6—C1	−0.7 (2)	C17—N16—C15—N14	−178.51 (11)
N2—C1—C6—C5	0.65 (18)	C17—N16—C15—C20	3.62 (18)
N7—C1—C6—C5	−179.63 (12)	C11—N14—C15—N16	145.45 (11)
C1—N7—C8—C13	−139.63 (12)	C11—N14—C15—C20	−36.68 (18)
C1—N7—C8—C9	44.73 (17)	C15—N16—C17—C18	−1.0 (2)
C13—C8—C9—C10	2.02 (17)	N16—C17—C18—C19	−2.2 (2)
N7—C8—C9—C10	177.69 (11)	C17—C18—C19—C20	2.9 (2)
C8—C9—C10—C11	−0.71 (18)	C18—C19—C20—C15	−0.5 (2)
C9—C10—C11—C12	−1.57 (18)	N16—C15—C20—C19	−2.90 (19)
C9—C10—C11—N14	175.07 (11)	N14—C15—C20—C19	179.36 (12)

### Hydrogen-bond geometry (Å, °)

**Table:**

*D*—H···*A*	*D*—H	H···*A*	*D*···*A*	*D*—H···*A*
N7—H7N···N16^i^	0.90	2.25	3.1423 (13)	175
N14—H14N···N2^ii^	0.90	2.12	3.0141 (14)	175

Symmetry codes: (i) *x*, *y*, *z*+1; (ii) *x*, *y*, *z*−1.
